# Enhancement of a Magnetically Controlled Cathodic Arc Source for the Deposition of Multi-Component Hard Nitride Coatings

**DOI:** 10.3390/ma18102276

**Published:** 2025-05-14

**Authors:** Van-Tien Tang, Yin-Yu Chang, Yi-Ru Chen

**Affiliations:** Department of Mechanical and Computer-Aided Engineering, National Formosa University, Yunlin 63201, Taiwan; tangtienthoha@gmail.com (V.-T.T.); joeae85@gmail.com (Y.-R.C.)

**Keywords:** cathodic arc deposition, magnetic field, TiAlNbSiN, AlCrSiN, mechanical property

## Abstract

The creation of coatings by the cathodic arc evaporation method has outstanding advantages: these coatings are highly durable and wear-resistant, especially since the method has an intense ionization process and the atoms can penetrate deep into the surface substrates, resulting in excellent adhesion. Furthermore, this approach provides precise control over the chemical composition and thickness of the coating, ensuring consistent quality across the entire surface. However, uneven evaporation and ejection of molten metal droplets from the cathode during cathode arc deposition produce particles and droplets, resulting in an uneven coating surface. This study presents a new design for a magnetically controlled cathode arc source to effectively reduce particles and droplets during the cathodic arc deposition of multi-component alloy targets for nitride-based hard coatings. The study compares the performance of a new source with a conventional magnetic-controlled arc source for depositing TiAlNbSiN and AlCrSiN films. In the conventional source, the magnetic field is generated by a permanent magnet (PM), whereas in the new source, it is generated and controlled using an electromagnet (EM). Both films are produced using multi-component alloy targets (TiAlNbSi and AlCrSi) with identical composition ratios. The plasma characteristics of the two different arc sources are investigated using an optical emission spectrometer (OES), and the surface morphology, structural characteristics, deposition rate, uniformity, and surface roughness (Sa) are examined using scanning electron microscopy (SEM). When the EM was applied to have high plasma density, the hardness of the TiAlNbSiN film deposited with the novel arc source measured 31.2 ± 1.9 GPa, which is higher than that of the PM arc source (28.3 ± 1.4 GPa). In contrast, the AlCrSiN film created using a typical arc source exhibited a hardness of only 25.5 ± 0.6 GPa. This lower hardness may be due to insufficient ion kinetic energy to enhance stress blocking and increase hardness, or the presence of the h-AlN phase in the film, which was not detected by XRD. The electromagnet arc source, with its adequate ion bombardment velocity, facilitated a complementary effect between grain growth and stress blocking, leading to a remarkable hardness of 32.6 ± 0.5 GPa.

## 1. Introduction

One of the important manufacturing processes to produce various part geometries for different engineering materials is machining [1]. In this manufacturing process, the productivity and quality of machined parts are significantly dependent on the use and wear of those cutting tools [2]. However, working under high-impact forces and temperatures is a major challenge for cutting tools [1,2]. For that reason, there is a need for solutions that can improve tool performance, and surface treatments have been shown to significantly improve cutting tool performance while working under harsh working conditions [3,4,5]. Some previous research shows that coated tools exhibit better performance and longer life than uncoated tools [6,7]. The purpose of applying these coatings to the surface of the cutting tool is to enhance the structural, mechanical, and tribological properties of the coated tool material [8,9,10].

Recent studies have shown that TiN exhibits suitable characteristics for tribological coatings, including high hardness, strong adhesion to metallic surfaces, and good chemical stability [11]. Additionally, TiN coatings offer excellent high-temperature and corrosion resistance [12]. In addition to TiN coatings, CrN coatings serve as a TiN coating replacement due to their almost identical characteristics [13]. Even though both coatings have acceptable mechanical qualities, the coating will oxidize when subjected to extreme working conditions during the cutting process. This will have a negative impact on the pace at which the cutting is performed. J.M Castanho and M.T Vieira showed that the oxidation problem is eliminated with the formation of an Al_2_O_3_ layer on the surface at high temperatures when Al is added to TiN and CrN coatings [14]. Adding silicon (Si) to TiAlN and CrAlN coatings improves performance. Research indicates that AlCrSiN coatings provide greater hardness, surface roughness, and wear resistance than AlCrN coatings [15,16,17]. Vennemann et al. demonstrated that TiAlSiN coatings exhibit significantly enhanced thermal stability and hardness compared to TiAlN coatings [18]. Additionally, TiAlSiN coatings provide excellent long-term oxidation resistance [19].

Selecting high-performance coating ingredients is essential, but the deposition process is equally crucial in defining the coating’s structure and properties. Cathodic arc evaporation is a highly effective method for applying multicomponent hard coatings, delivering exceptional wear resistance, heat resistance, tribological benefits, and attractive ornamental qualities. This approach ensures superior performance and durability in coatings. Indeed, highly ionizing discharges enable fine control of ion–surface interactions during both the ion etching and film deposition processes [20]. However, the coating surface created by the cathodic arc will also generate particles and droplets, resulting in flaws such as an uneven film surface [21]. Several arc sources have been developed to decrease particles and droplets on the coated surface, including the use of magnetic or electromagnetic fields to control the arc’s travel [22]. Ana B.B. Chaar et al. found that when using a combination of permanent magnets and electromagnetic coils, as well as adjusting the current of the electromagnetic coils to 0–2 A to deposit AlTiN, appropriate parameters can effectively reduce particles [23]; K.J. Feng et al. used a similar combination and adjusted the current of the electromagnetic coils to 0–5 A to deposit ta-C films, and achieved similar results [24]. It is clear that the adjustment of the electromagnetic field is essential for the results of cathode arc deposition.

In this experiment, the EM arc source and the PM arc source in the plating of multi-element alloy targets were contrasted. The current setting refers to the experimental parameters of 0~2 A studied by Ana B.B. Chaar et al. [23]. However, when the coil current is 2 A, the arc is easily broken during the film deposition process, which can easily reduce the experimental accuracy. Therefore, the coil current is appropriately adjusted to stabilize the target during the film deposition process. Two target materials containing silicon (Si) components were chosen in this study: Ti_44_Al_40_Nb_12_Si_4_ and Al_60_Cr_30_Si_10_. An optical plasma spectrometer (OES) was used to study and compare the plasma properties of the two multi-element alloy targets by altering the distribution of the magnetic field on the target surface. The arc motion pattern was also observed. The TiAlNbSiN and AlCrSiN nitride single-layer hard films were plated, and their microstructures, mechanical properties, and other film-related analytical tests were investigated to determine which magnetic field configuration could reduce particles and improve film performance, thereby improving the cathodic arc-evaporated film quality.

## 2. Experimental Details

### 2.1. Coating Deposition

**Figure 1:**
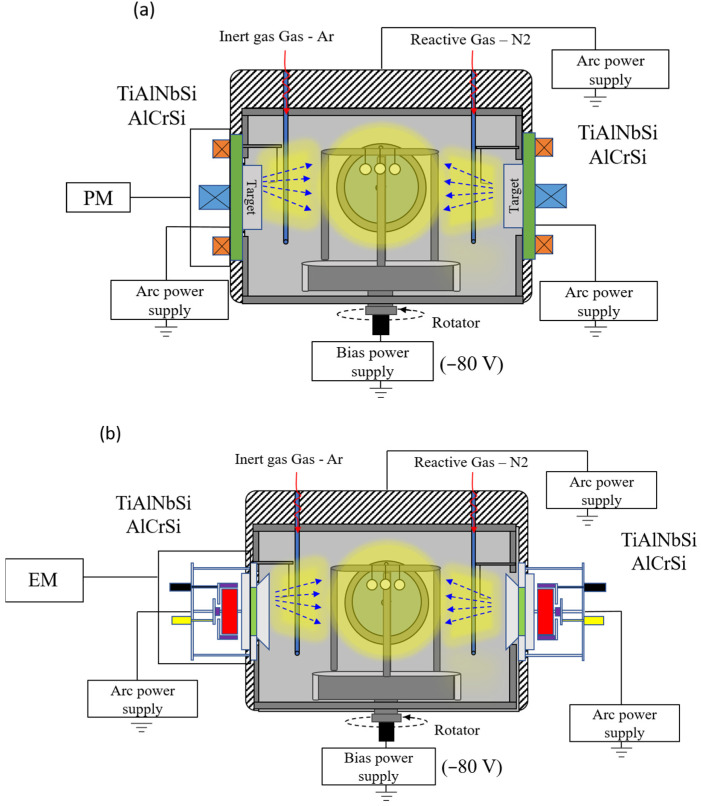
Schematic diagram of cathode arc deposition system with different magnetron arc sources: (**a**) permanent magnetic (PM)-controlled arc source and (**b**) new electromagnetic (EM)-controlled arc source.

In this experiment, TiAlNbSiN and AlCrSiN coatings were deposited on P-type (100) single-crystal silicon wafers and tungsten carbide (WC-Co) samples (20 mm in diameter and 4 mm thick) by a cathodic arc deposition system with a PM arc source and an EM arc source. The WC-Co specimens underwent a thorough polishing process, achieving a mirror finish with a surface roughness (Ra) of about 50 nanometers, enhancing their aesthetic and functional qualities. To remove any remaining impurities, the polished specimens were immersed in a supersonic cleaning bath of ethanol for 15 min, then 15 min in acetone, using high-frequency sound waves to create bubbles for effective cleansing. Afterwards, the pristine specimens were securely placed in specialized rotating substrate holders within the deposition apparatus, ensuring stable support and uniform coating during the subsequent processes. Figure 1 depicts two deposition chambers. Figure 1a displays the deposition chamber with a PM arc source, whereas Figure 1b depicts the novel arc source deposition chamber. In the novel arc source, the magnetic field is controlled by an electromagnet, in which the current intensity is controlled at 1A.

To determine this optimal setting, preliminary experiments were conducted at different electromagnet currents (0 A, 1 A, and 1.75 A). The results showed that operating at 1 A produced the most stable arc behavior, the highest coating hardness, and the lowest droplet density. Therefore, the current intensity of 1 A was selected for all subsequent depositions to achieve enhanced plasma control and coating quality.

Both deposition devices had two sets of targets and an arc power supply. In addition, the used targets are pure chromium (Cr), a multi-alloy target of titanium aluminum niobium silicon target (Ti:Al:Nb:Si = 44:40:12:4 at.%) for TiAlNbSiN, and an aluminum chromium silicon target (Al:Cr:Si = 60:30:10 at.%) for AlCrSiN.

**Figure 2:**
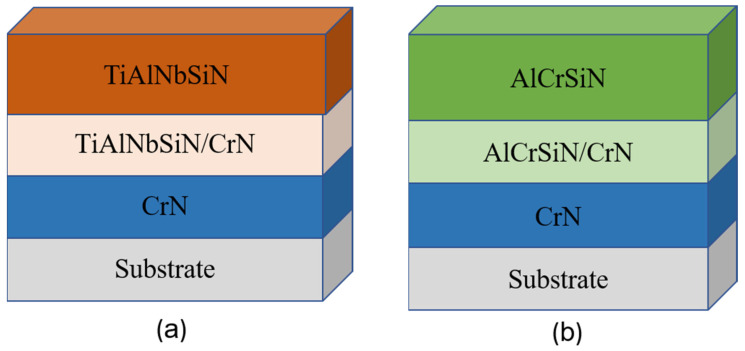
Schematic diagram of coating structures: (**a**) TiAlNbSiN and (**b**) AlCrSiN.

Table 1 shows the deposition parameters in detail. The workpiece is connected to a negative bias power supply and is located roughly 363 mm away from the samples. The nitride coatings are created via the deposition of argon (Ar) and nitrogen (N_2_). To begin the coating deposition procedure, the vacuum chamber was warmed to 300 °C and pumped down to less than 1 × 10^−3^ Pa. The samples were subsequently cleaned by ion bombardment with Ar ions at −800 V in an Ar environment of 0.8 Pa for 30 min. The first layer consists of a thin Cr layer covered with a pure chromium (Cr) target to improve substrate–film adhesion. The second layer contains a nitrogen reaction gas that forms a nitride film CrN, which improves the film’s adhesion. The third layer is a transition layer, which combines TiAlNbSiN and AlCrSiN with CrN to generate TiAlNbSiN/CrN and AlCrSiN/CrN, respectively, to maintain film grain growth continuity. Finally, the top layer is coated with TiAlNbSiN and AlCrSiN with controlled magnetic fields to investigate how different magnetic fields affect the properties of the coatings. Figure 2a,b show a schematic depiction of the film structure.

### 2.2. Coating Characterization

The chemical composition of the deposited coatings was determined with a JEOL JXA-iHP200F electron probe microanalyzer (EPMA) (JEOL Inc., Tokyo, Japan). The coatings’ shattered morphologies were investigated with a field emission scanning electron microscope (FESEM, JSM-7610Plus, JEOL Inc., Tokyo, Japan). The crystal structures were studied using an X-ray diffractometer (XRD, D8 Discover, Bruker Inc., Billerica, MA, USA) with a Cu Kα (wavelength λ = 0.15406 nm) source at 40 kV and 40 mA at a grazing angle of 2°. The scanning angle spanned from 20° to 90°, with a specific scanning step of 0.02° and a scanning speed of 1° per minute. Furthermore, during the deposition process, an optical emission spectrometer (OES) was employed to investigate the plasma ion state generated by various magnetron arc sources in the deposition process. A plasma spectrometer was used to determine the state of metal ions evaporated by various magnetron arc sources, such as valence and ionic strength. Furthermore, changes in the element intensity of multi-element alloy targets were detected in various magnetron arc sources, and the differences between the two were examined. It is predicted that by using a spectrometer, the intensity variations of the plasma state may be instantaneously comprehended during the production process. This experiment was carried out using the GEI MarsHS2000+ (GEI Industrial, Montgomeryville, PA, USA). In this experiment, the arc movement during the plasma analysis was observed through the observation window of the cathode arc evaporation coating machine. Given the brief arc reaction time on the target surface, more advanced photographic equipment was necessary. Consequently, a digital camera was securely positioned near the window, utilizing the ProCam 8 software to capture images of the arc’s movement on the target surface.

In this study, the adhesion strength between the film and the substrate was evaluated using a Rockwell hardness tester (HR-200, Mitutoyo Co., Kawasaki, Japan) with an applied load of 60 kg, following the international standard ISO 26443:2008 [25]. The Rockwell hardness test involved applying a static load and observing the resulting indentation morphology using an optical microscope to determine the adhesion level based on the extent of cracking or delamination around the indent. In addition, a nanoindentation tester was employed to measure the coatings’ mechanical properties, including hardness and Young’s modulus. The instrument consists of a tiny diamond or carbide conical tip, which is loaded on the surface of the test piece through a very precise force sensor and measures the applied force. During this process, the tip creates a small indentation in the material surface, the shape and size of which depend on the tip shape and the force applied. The shape and size of the indentation can be used to calculate mechanical properties, such as the material’s hardness and Young’s modulus. To prevent the measured value from being affected by the hardness of the substrate itself, the indentation depth should not exceed one-tenth of the film thickness [26]. The measured hardness and Young’s modulus data can be used to calculate the resistance to plastic deformation H^3^/E^2^. The larger the value, the better the film’s resistance to plastic deformation, and the less likely it is to deform due to stress [27].

## 3. Results and Discussion

### 3.1. Chemical Composition

Table 2 displays the TiAlNbSiN and AlCrSiN coatings’ chemical concentrations when coated with PM and EM, which were determined by EPMA. There is no significant difference in the elemental composition of coatings produced using either the old or new cathodic arcs. This indicates that switching between these types of cathodic arcs has a minimal effect on the content of the constituent elements in the coatings. The nitrogen content of all four coatings is approximately 50 atomic percent (at.%), which plays a significant role in the formation of nitride compounds. This relatively elevated nitrogen concentration in the coatings can be attributed to the high deposition pressure maintained at 3.33 Pa. Both the TiAlNbSiN (PM) and TiAlNbSiN (EM) coatings contain approximately 24 atomic percent (at.%) titanium (Ti). In contrast, the niobium (Nb) concentration is modest, ranging between 7.8 and 7.9 at.%. The aluminum (Al) concentration ranges from 16.8 to 17.1 at.%, whereas the silicon (Si) percentage is negligible, remaining below 1 at.%. This low silicon percentage is due to the alloy targets utilized, which only contain around 4 at.% silicon. In comparison, the AlCrSiN coatings, both PM and EM types, have a significantly higher aluminum content, ranging from 28.3 to 28.8 at.%. Additionally, the silicon content in these coatings falls between 3.4 and 3.8 at.%, which is considerably higher than that found in the TiAlNbSiN coatings.

### 3.2. Microstructure Characterization

**Figure 3:**
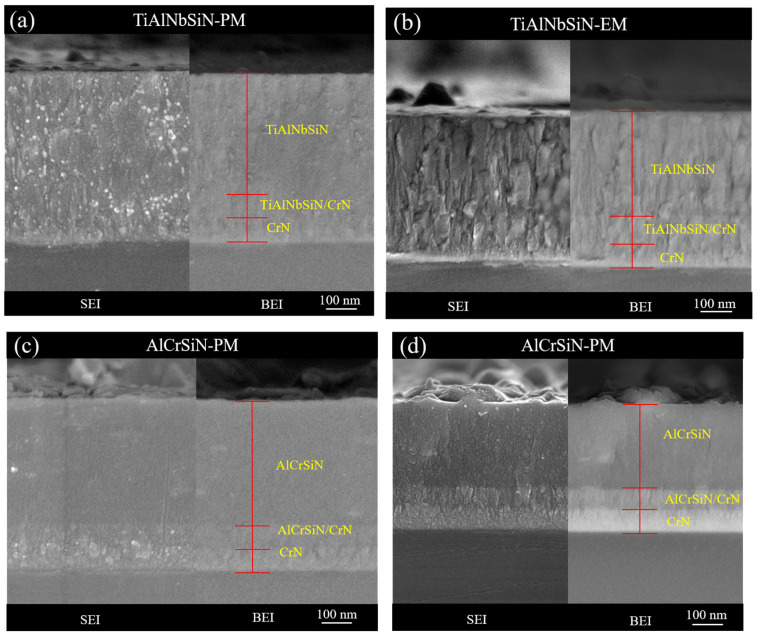
SEI-SEM and BEI-SEM images of the cross-sectional morphology of TiAlNbSiN and AlCrSiN coatings under different magnetic field configurations: (**a**) TiAlNbSiN-PM, (**b**) TiAlNbSiN-EM, (**c**) AlCrSiN-PM, and (**d**) AlCrSiN-EM.

The film microstructures were observed using a high-resolution field emission scanning electron microscope (FESEM) to explore the microstructural changes of TiAlNbSiN and AlCrSiN thin films under different magnetic configurations. In Figure 3a, it can be observed that the cross-section of the TiAlNbSiN film deposited by the PM arc source has higher coating thickness than that of the same coating deposited by EM, and the addition of Si refines the grains and inhibits the growth of columnar crystals [28]. The TiAlNbSiN film deposited by the EM arc source has a more obvious columnar crystal structure, which can be seen in Figure 3b. It is speculated that due to the magnetic field distribution of the EM arc source and the high ionization rate of the plasma, the high-energy ions increased the recrystallization rate [29]. The added Si component is not enough to inhibit grain growth, resulting in a more obvious columnar crystal structure. In the AlCrSiN film deposited by the PM arc source in Figure 3c, it can also be observed that it has higher coating thickness than that of the same coating deposited by EM, and the addition of Si refines the grains and inhibits the growth of columnar crystals, resulting in a dense structure [30]. Figure 3d depicts the AlCrSiN coating generated with the EM arc source, which has a more visible columnar crystal structure. Both the TiAlNbSiN and AlCrSiN coatings displayed a similar trend in deposition rate and coating growth, which showed a lower deposition rate and a more visible columnar crystal structure observed in the coatings deposited by EM. 

**Figure 4:**
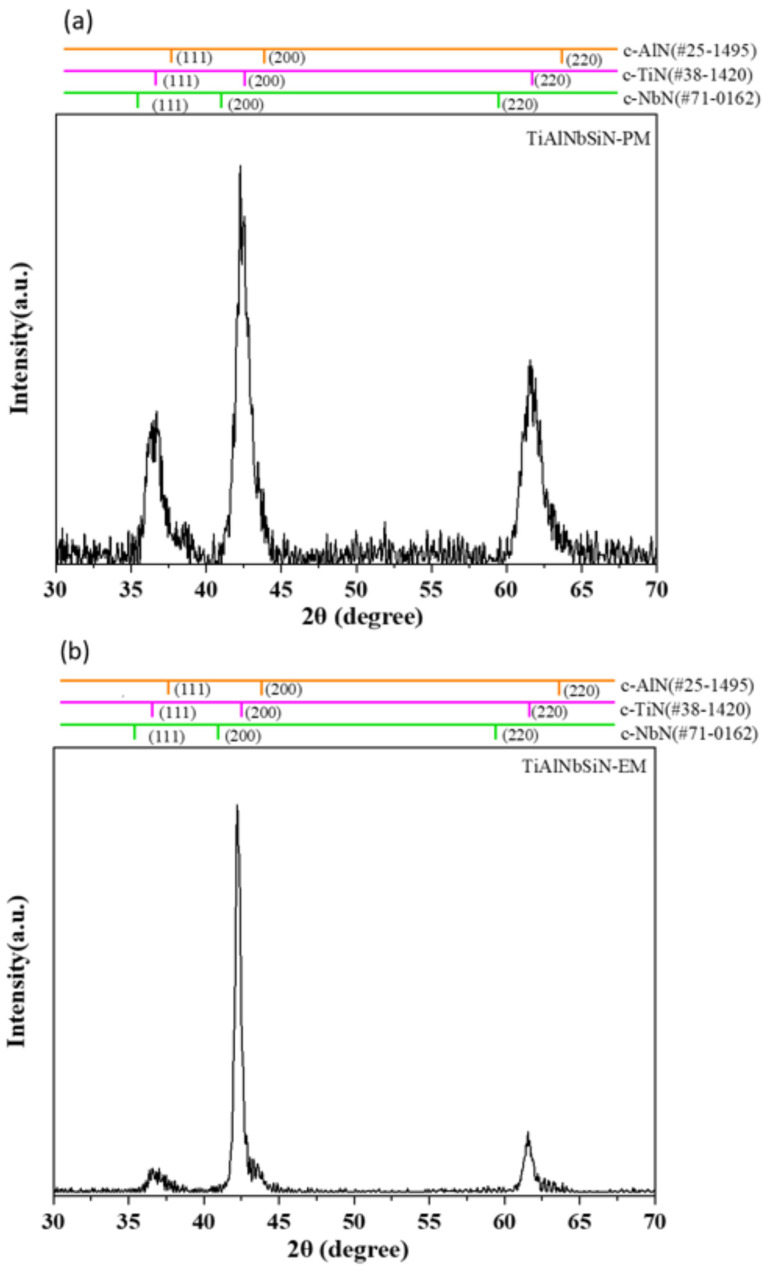
X-ray diffraction patterns of TiAlNbSiN coatings under different magnetic field configurations: (**a**) TiAlNbSiN-PM, (**b**) TiAlNbSiN-EM.

**Figure 5:**
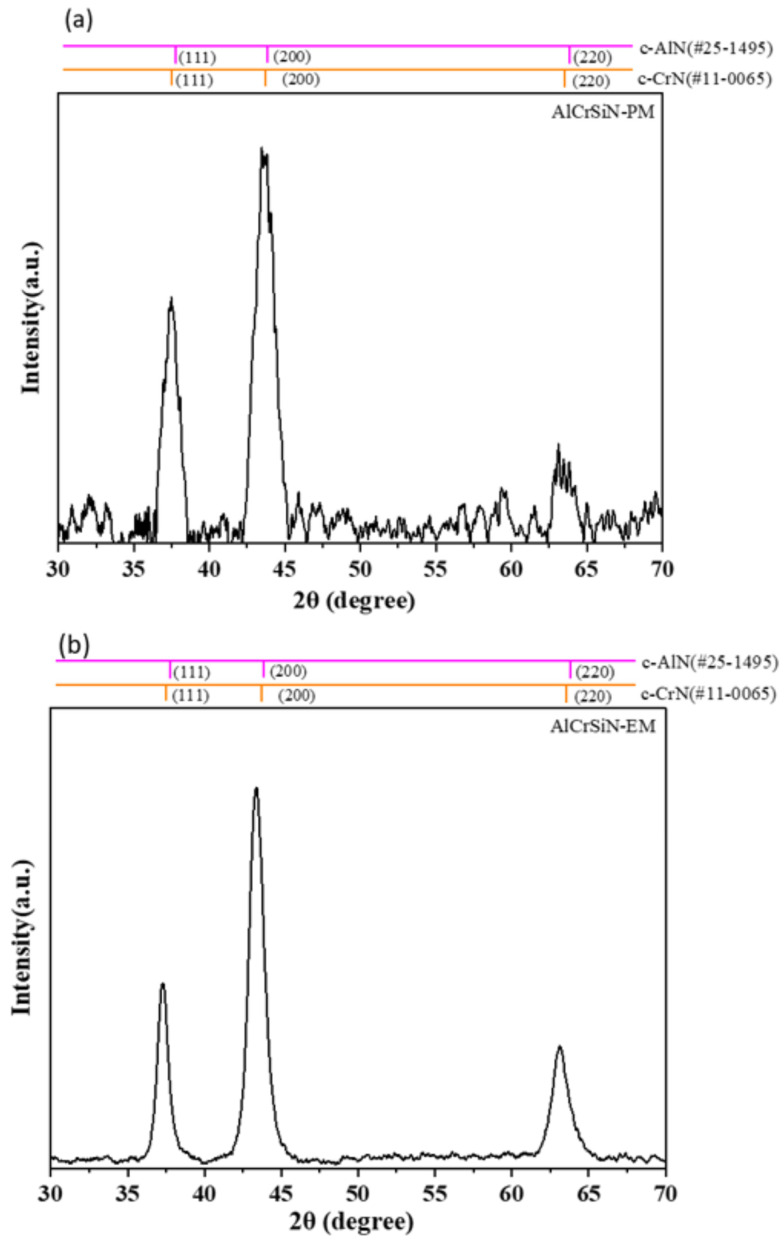
X-ray diffraction patterns of AlCrSiN coatings under different magnetic field configurations: (**a**) AlCrSiN-PM and (**b**) AlCrSiN-EM.

XRD patterns of all coatings are shown in Figure 4 and Figure 5, while Figure 4a,b show the X-ray diffraction analysis of TiAlNbSiN films deposited by the PM arc source and the new EM arc source. TiAlNbSiN films mainly have (111), (200), and (220) crystal planes. After checking according to the JCDPF database, its main peak position is quite close to TiN (JCDPF#38-1420) and between NbN (JCDPF#71-0162) and AlN (JCDPF#25-1495). All three planes are FCC face-centered cubic B1-NaCl crystal structures, so there is a solid solution phenomenon [31]. Since the Si component is small in the film, it may exist in the amorphous state of SiN_x_. However, it can still be observed that the preferred direction is (200) due to the addition of Si, and the signal intensity of the (200) crystal plane of the EM arc source is much higher than that of the PM arc source (200). The Si component in the TiAlNbSiN film could originally inhibit grain growth [28], but the Si content is not enough to suppress defects caused by high-energy ion bombardment of the EM arc source and increase the recrystallization rate [32,33]. Figure 5a,b display the X-ray diffraction analysis diagrams of AlCrSiN films deposited using both PM and EM arc sources. The AlCrSiN film primarily exhibits crystal planes at (111), (200), and (220). According to the JCPDS database, the main peak is observed between CrN (JCPDS#11-0065) and AlN (JCPDS#25-1495). Both compounds adopt a face-centered cubic (FCC) B1-NaCl crystal structure, which leads to similar solid solution phenomena. The inclusion of silicon leads to a preferred orientation of (200) [34], with silicon present in the film as amorphous SiN_x_. However, the AlCrSiN film did not exhibit a stronger (200) crystal plane signal typical of the EM arc source deposition, which was significantly more pronounced than that observed in TiAlNbSiN with an EM arc source. The primary distinction between AlCrSiN and TiAlNbSiN lies in the target material used for the AlCrSiN film, which possesses a higher silicon content. This increased silicon allows for the production of more amorphous SiN_x_, effectively mitigating defects that can arise from high-energy ion bombardment.

### 3.3. Magnetic Field Distribution and Arc Type

**Figure 6:**
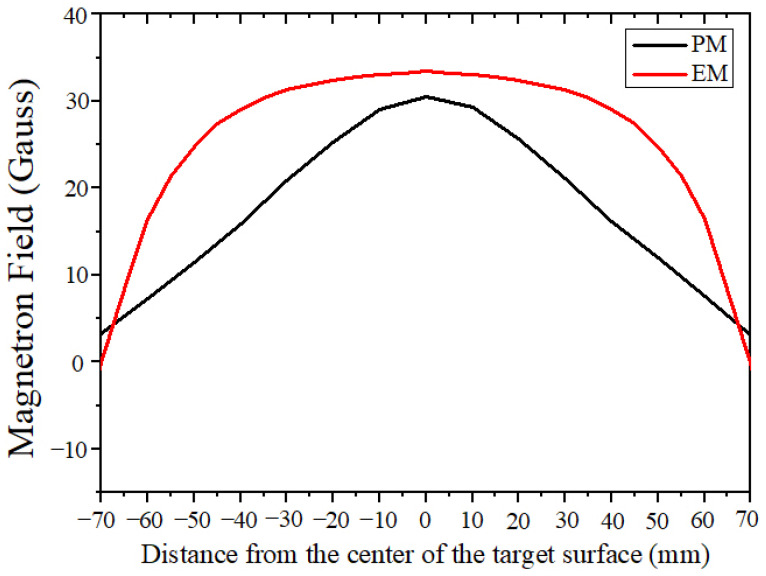
Magnetic field distribution on the target surface in PM and EM arc source configurations.

In this experiment, a Gauss meter was used to measure the magnetic field distribution of the EM arc source. The results were compared with the PM arc source. As shown in Figure 6, it was found that the EM arc source has a peak magnetic field strength of around 35 Gauss, while the PM arc source only has a peak of 30 Gauss. Using the EM arc source, the magnetic field distribution curve showed an overall uniform and enhancement trend, and the target surface was subjected to a closed co-directional magnetic field. The magnetic field distribution curve when using the PM arc source tends to concentrate at the center of the target. This phenomenon can be explained by the fact that when the current in the electromagnetic coil is set to 1A, the alternating magnetic field strength (AMF) also increases, which alters the distribution of the magnetic field on the cathode surface [35].

In the case of a permanent magnet, the magnetic field lines are primarily parallel to the cathode surface, leading to random movement of the cathode spot, which is mainly located about 20 mm from the center of the cathode. However, when employing an electromagnet with a coil current of 1A, the magnetic field lines near the cathode edge direct themselves toward the ring anode, resulting in an increased magnetic flux density in that area [23]. Consequently, the cathode spot becomes confined closer to the cathode edge (approximately 50 mm from the center) and tends to move in a more stable orbit.

**Figure 7:**
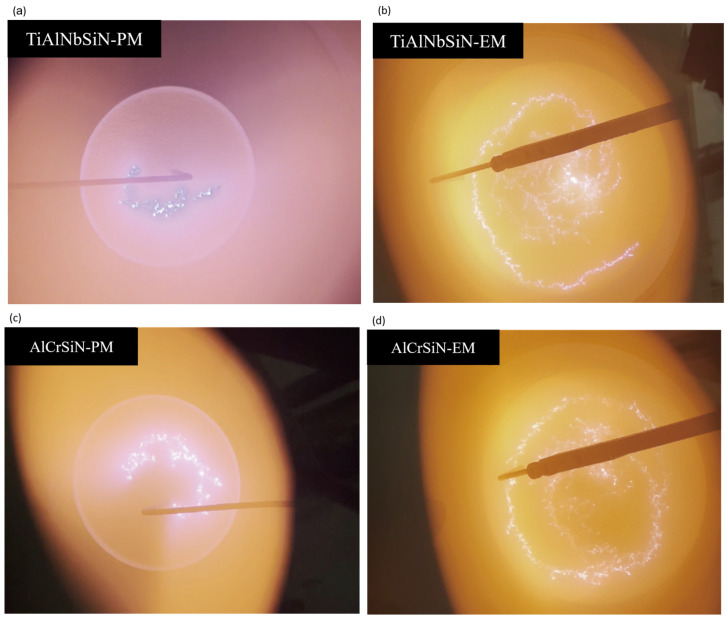
Arc trajectory profiles on the TiAlNbSi and AlCrSi target surfaces during the deposition of TiAlNbSiN and AlCrSiN thin films using PM and EM arc source configurations. (**a**) TiAlNbSiN-PM; (**b**) TiAlNbSiN-EM; (**c**) AlCrSiN-PM; (**d**) AlCrSiN-EM.

Figure 7 shows the arc trajectory profiles on the TiAlNbSi and AlCrSi target surfaces during the deposition of TiAlNbSiN and AlCrSiN thin films using PM and EM arc source configurations. During the deposition process of TiAlNbSiN and AlCrSiN thin films using PM arc sources, it can be seen in Figure 7a,c that the arc distribution density on the target surface is low, and the arc is short and rough but moves rapidly. The arc density of the new EM arc source in Figure 7b,d is much higher than that of the PM arc source. With the EM arc source, the arc shape is the same as the result of W.C. Lang et al.’s study [35], which is evenly distributed on the target surface in a spiral shape. In addition, the spiral arc in the EM configuration was more refined and moved faster, and the overall plasma density might be improved.

### 3.4. Optical Emission Spectroscopy (OES)

**Figure 8:**
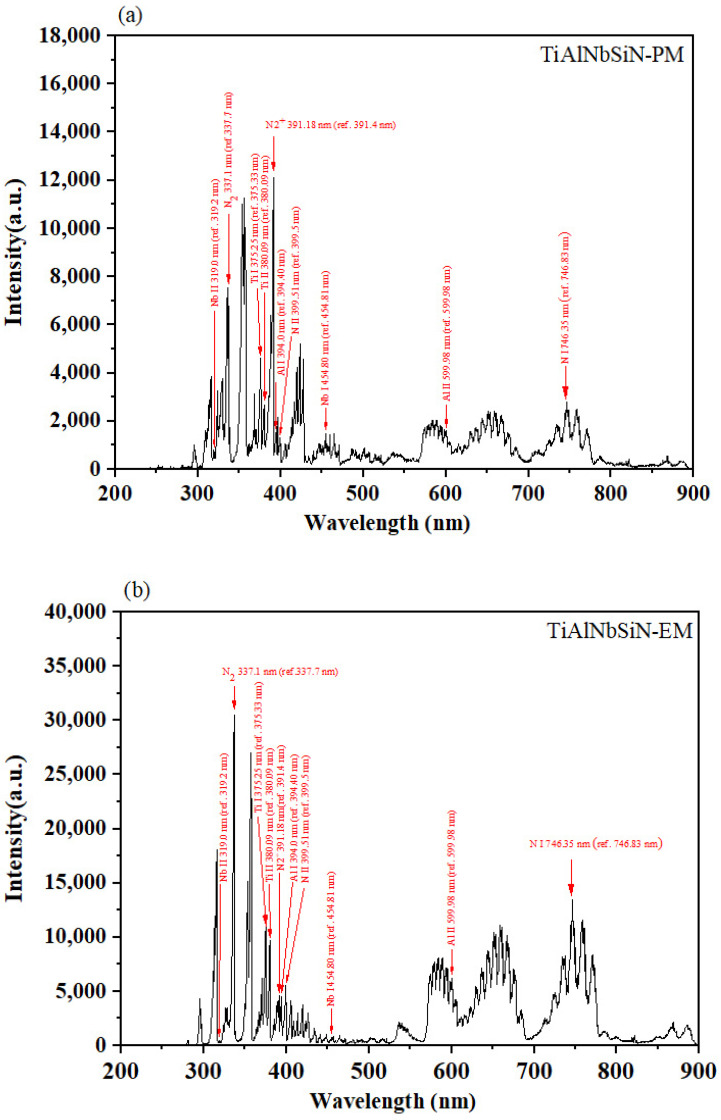
OES spectra during the deposition of TiAlNbSiN with PM and EM arc source configurations. (**a**) TiAlNbSiN-PM; (**b**) TiAlNbSiN-EM.

**Figure 9:**
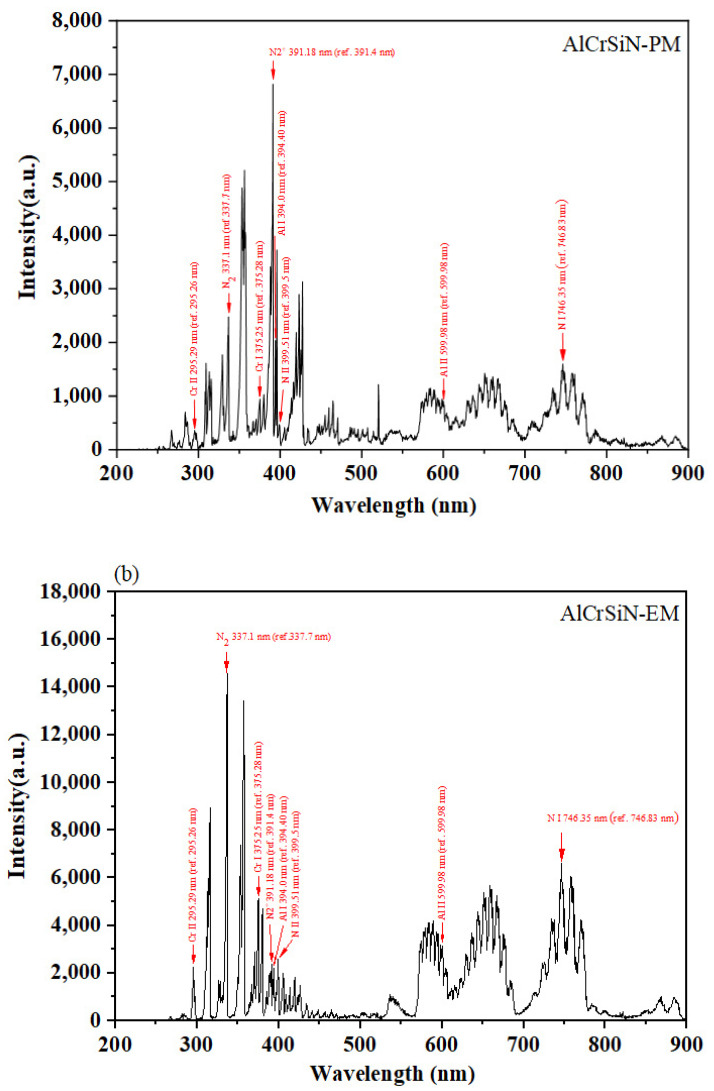
OES spectra during the deposition of AlCrSiN with PM and EM arc source configurations. (**a**) AlCrSiN-PM; (**b**) AlCrSiN-EM.

Figure 8a,b illustrate the optical emission spectra during the deposition of TiAlNbSiN with PM and EM arc source configurations, respectively. These spectra revealed emissions from several species, such as Ti atoms at 375.3 nm, Ti^+^ ions at 380.1 nm, N atoms at 746.4 nm, N^+^ ions at 399.5 nm, N_2_ molecules at 337.1 nm, N_2_^+^ ions at 391.18 nm, Nb atoms at 454.8 nm, Nb^+^ ions at 319.0 nm, Al atoms at 394.0 nm, and Al^+^ ions at 600.0 nm. Figure 9a,b show the OES spectra of the AlCrSiN films, with emissions from Cr atoms (375.3 nm), Cr^+^ ions (295.3 nm), N atoms (746.4 nm), N^+^ ions (399.5 nm), N_2_ molecules (337.1 nm), N_2_^+^ ions (391.2 nm), Al atoms (394.0 nm), and Al^+^ ions (600.0 nm). The data lines are from the NIST database [36], and the N_2_ molecule spectral lines are from A. Qayyum et al. [37]. There are no Si peaks detected in either film’s spectra, likely due to high-intensity emissions from other elements and the low Si content.

**Figure 10:**
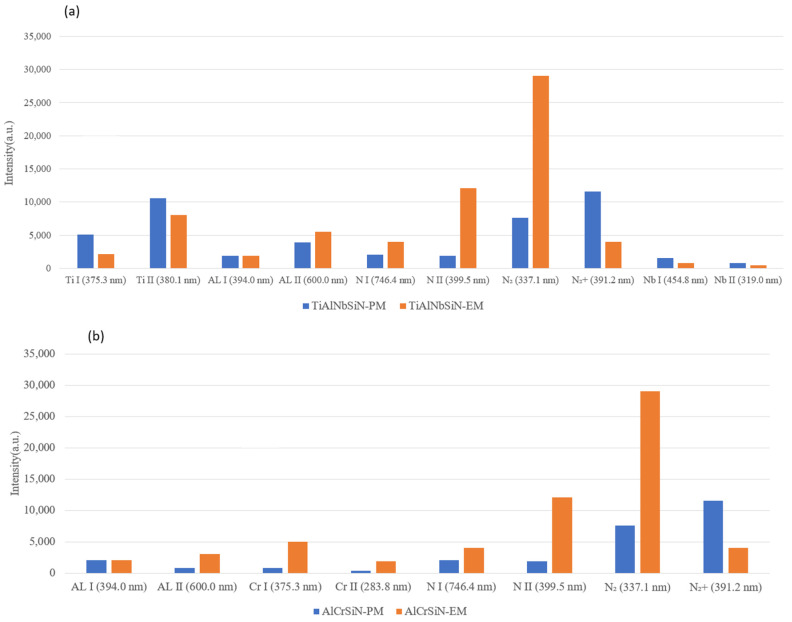
OES spectra signal intensities of the excited plasma during the deposition of (**a**) TiAlNbSiN and (**b**) AlCrSiN with PM and EM arc source configurations.

The comparison of the OES spectra for TiAlNbSiN and AlCrSiN films, illustrated in Figure 8 and Figure 9, demonstrates that the overall average signal intensity from the EM arc source is considerably greater than that from the PM arc source. Furthermore, Section 3.3 highlights that the emitted light during the deposition of TiAlNbSiN and AlCrSiN films using the EM arc source shares a similar color and exhibits higher brightness. By analyzing the spectral peak signals in relation to available databases and literature, we can conclude that the emitted light primarily comes from nitrogen. This phenomenon occurs because, under certain strengths of magnetic fields, the likelihood of collisions between high-energy electrons and nitrogen molecules increases. The circular motion of the electrons, influenced by the magnetic field, significantly enhances the likelihood of gas dissociation. Furthermore, the circular motion of electrons allows high-energy electrons to ionize the material evaporated from the cathode. Consequently, in Figure 10a,b, we observe that the signals for the atoms and ions generated by most elements in the EM arc source are enhanced.

### 3.5. Surface Characteristics

**Figure 11:**
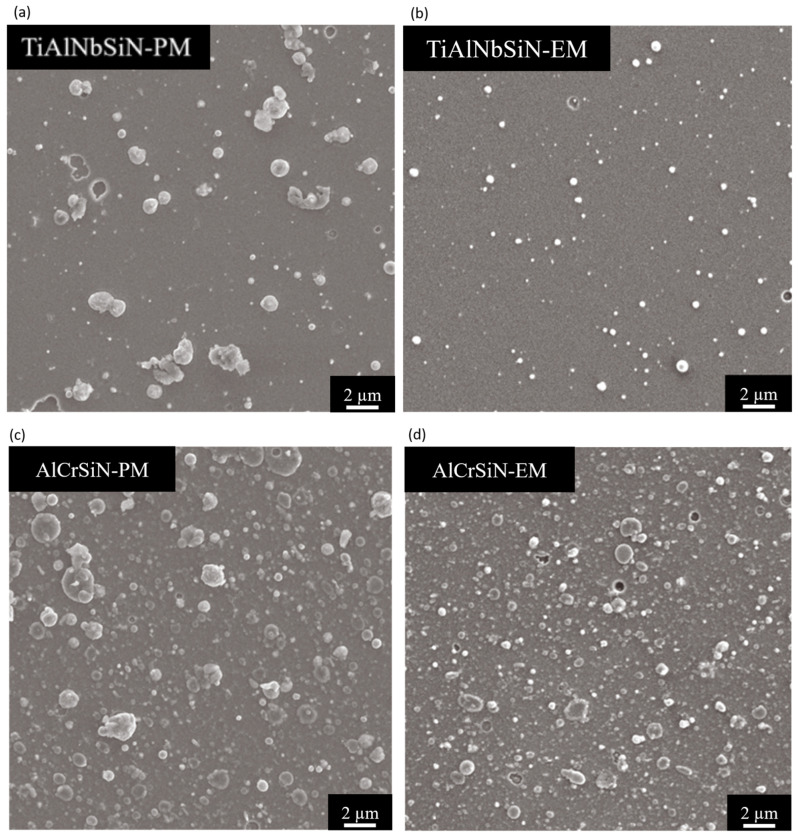
Surface morphology of the deposited TiAlNbSiN and AlCrSiN coatings using PM and EM arc source configurations: (**a**) TiAlNbSiN-PM, (**b**) TiAlNbSiN-EM, (**c**) AlCrSiN-PM, and (**d**) AlCrSiN-EM.

Figure 11 presents the surface morphologies of TiAlNbSiN and AlCrSiN films deposited using both PM arc and EM arc source configurations. From Figure 11a,b, it is evident that the diameter and number of TiAlNbSiN film particles deposited by the EM arc source are smaller compared to those deposited by the PM arc source. This difference occurs because the cathode arc spots of the PM arc source do not move as quickly as those of the EM arc source. As a result, the cathode point remains in one position for a longer duration, which leads to a larger molten pool area generated by the high-density current. This larger area tends to produce larger particles in greater quantities. Conversely, the EM arc source minimizes both the number and diameter of particles due to the rapid movement of the cathode point. Consequently, the EM arc source results in the smallest number and diameter of particles when applied to coat the material. The AlCrSiN film exhibited results similar to those of the TiAlNbSiN films. However, in Figure 11c,d, the size of the particles on the AlCrSiN film surface was noticeably larger, and their number increased, which was attributed to target poisoning [36,38]. AlCrSiN coatings have more macroparticles compared to TiAlNbSiN coatings primarily because the silicon (Si) concentration in the AlCrSi target is significantly higher at 10 atomic percent (at.%) compared to just 4 at.% in the TiAlNbSi target. Silicon possesses semiconductor properties, which disrupt the arc formation on the target. Additionally, aluminum (Al) has a much lower melting point than chromium (Cr), leading to the creation of larger macroparticles in greater quantities. In the AlCrSiN film produced with the EM arc source, we observe a scenario where the particle diameter is small, but the number of particles is large. This can be attributed to the rapid movement of the cathode point over the target surface, influenced by the magnetic field. Consequently, when comparing AlCrSiN films, even though the number of particles cannot be significantly reduced, we can consider the surface roughness they have.

Table 3 demonstrates that both TiAlNbSiN and AlCrSiN films deposited using the EM arc source exhibit reduced surface roughness (Sa) values. This improvement can be attributed to the magnetic field generated by the EM arc source, which accelerates the movement of the cathode point across the target surface. Consequently, the molten pool’s time and area are minimized, resulting in the formation of fewer and smaller particles that contribute to a smoother film surface compared to the PM arc source [35].

Figure 7 shows that for TiAlNbSiN films, the cathode point undergoes a rapid circular motion, which promotes a more uniform arc motion. This results in a reduced number and size of particles, contributing to superior surface flatness and optimized surface roughness. Conversely, while AlCrSiN films also benefit from the rapid circular motion of the cathode point with the EM arc source, the presence of target poisoning leads to a greater number of surface particles [36,38]. However, the significantly smaller particle size helps mitigate the height differences caused by these particles, ultimately resulting in reduced surface roughness.

### 3.6. Adhesion Strength and Mechanical Properties

**Figure 12:**
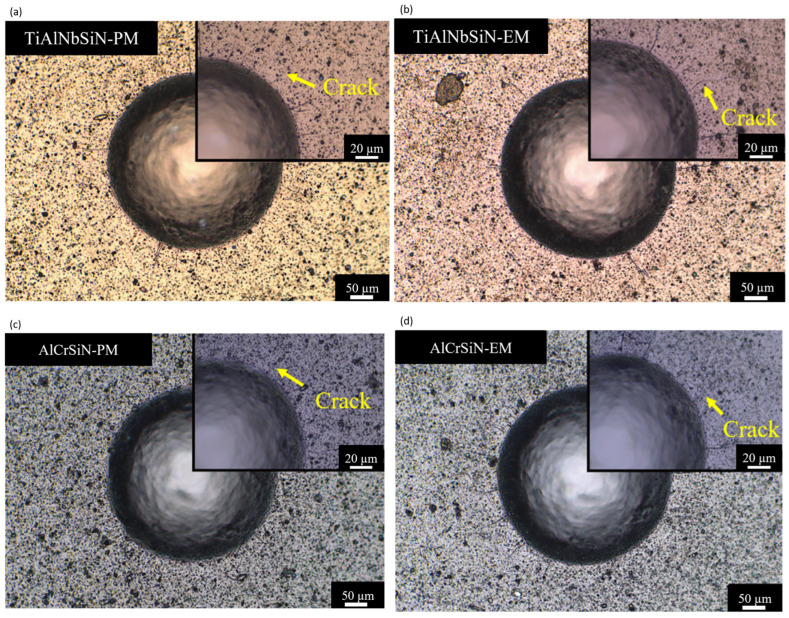
The SEM images of Rockwell indentation morphologies: (**a**) TiAlNbSiN-PM, (**b**) TiAlNbSiN-EM, (**c**) AlCrSiN-PM, and (**d**) AlCrSiN-EM.

To test the tungsten carbide sample, the ISO international standard specification (ISO 26443:2008) calls for using a 120° diamond indenter with a force of 60 kgf. The fractures and peeling surrounding the depression are next examined under an optical microscope. Figure 12 shows the findings for all films deposited using PM and EM arc sources. Both sets of films—those deposited with PM arc sources (shown in Figure 12a,c) and those with EM arc sources (shown in Figure 12b,d)—have some cracking around the indentation. According to the ISO international standard specification (ISO 26443:2008), the adhesion of these films is classified as Class 1. Based on these findings, all four films demonstrate Class 1, showing good coating adhesion strength, which can be attributed to the effective interlayer design in the film manufacturing process and the strong adhesion performance of the coatings.

The results of the nanoindentation test are shown below in Table 4. In the case of TiAlNbSiN films, the hardness and elasticity coefficients for those deposited using the PM arc source are measured at 28.3 ± 1.4 GPa and 372.4 ± 10.4 GPa, respectively. In contrast, the TiAlNbSiN deposited with the EM arc source shows a higher hardness value of 31.2 ± 1.9 GPa, while the elasticity coefficient was similar (371.0 ± 9.9 GPa). The coatings generated by the EM arc source show a noticeable enhancement in hardness when compared to those made from the PM source. This advancement is probably a result of the plasma produced by the EM arc source, which leads to ion bombardment that causes defects and alters the lattice within the crystal structure [39]. Consequently, this leads to an increase in hardness.

For AlCrSiN coatings, the hardness and elastic modulus of films deposited using the PM arc source are 25.5 ± 0.6 GPa and 274.2 ± 4.4 GPa, respectively. In contrast, the AlCrSiN deposited with the EM arc source exhibits higher hardness and elastic modulus values of 32.6 ± 0.5 GPa and 343.6 ± 8.6 GPa. The AlCrSiN film produced by the PM arc source shows lower hardness. According to a study by Bogdan Warcholinski et al. [40], increasing the silicon (Si) content in the target material leads to a higher volume of the amorphous phase SiN_x_, which results in a decrease in grain size. The PM arc source fails to generate sufficient stress to hinder dislocation due to inadequate ion kinetic energy, culminating in decreased hardness [41]. Moreover, even though X-ray diffraction (XRD) results did not reveal the presence of the h-AlN phase, numerous studies have indicated that this might also contribute to the observed decrease in hardness [30,42]. Obviously, both the TiAlNbSiN and AlCrSiN coatings deposited by EM exhibited higher hardness and H/E and H^3^/E^2^ values, which resulted from the higher plasma density and increased collisions between high-energy electrons and nitrogen molecules, and therefore enhanced the mechanical properties.

## 4. Conclusions

This study presents a comprehensive investigation into the use of both PM-controlled and EM-controlled arc sources for the deposition of two hard nitride coatings, TiAlNbSiN and AlCrSiN, utilizing multi-element alloy targets. By comparing these two arc sources, the research provides an in-depth analysis of the differences in plasma behavior during the deposition process.

A significant contribution of this work is the implementation and evaluation of a newly developed electromagnetically (EM) controlled arc source, which offers enhanced control over arc motion, plasma distribution, and film uniformity compared to the conventional permanent magnet (PM) system. The EM configuration generates a unidirectional closed magnetic field that promotes rapid, spiral arc motion and improved plasma ionization, resulting in marked improvements in film morphology and mechanical performance.

Experiments conducted at an EM coil current of 1A yielded the most stable arc behavior, the strongest plasma ionization, and the highest coating quality, thereby confirming the effectiveness of arc control at this setting. The results indicate that the 1A configuration optimally balances energy input and coating enhancement, serving as a practical reference for future applications.

Key findings reveal significant differences in surface morphology, microstructure, and mechanical properties of films deposited using EM versus PM arc sources. Coatings produced with the EM arc source demonstrated higher hardness, better crystallinity, reduced droplet formation, and more uniform surface textures, as confirmed by SEM, XRD, and nanoindentation analyses.

This study offers original and valuable insights into arc control strategies and lays a strong technical foundation for the industrial adaptation of EM arc sources in the deposition of multi-component hard nitride coatings.

## Figures and Tables

**Table 1:** Thin film process parameter table of the multi-element alloy target.

	Parameter
Arc Source	Permanent magnet arc source Electromagnet arc source
Target (at.%)	Ti_44_Al_40_Nb_12_Si_4_ Al_60_Cr_30_Si_10_
Reactive Gas	N_2_
Work Pressure	3.33 Pa
Target Current (A)	80
Bias Voltage (V)	−80
Temperature (°C)	300~400
Rotation Speed (rpm)	2

**Table 2:** Chemical composition of the investigated coatings.

Coatings	Chemical Composition (at.%)					
	N	Al	Ti	Nb	Si	Cr
TiAlNbSiN-PM	49.8 ± 0.7	16.8 ± 0.5	24.8 ± 0.3	7.8 ± 0.3	0.8 ± 0.1	-
TiAlNbSiN-EM	50.1 ± 0.5	17.1 ± 0.3	24.0 ± 0.2	7.9 ± 0.2	0.9 ± 0.1	-
AlCrSiN-PM	49.6 ± 0.2	28.3 ± 0.4	-	-	3.8 ± 0.4	18.3 ± 0.1
AlCrSiN-EM	50.6 ± 0.3	28.8 ± 0.5	-	-	3.4 ± 0.3	17.2 ± 0.1

**Table 3:** Average measured values of surface roughness of AlTiNbSiN and AlCrSiN films.

Coatings	TiAlNbSiN-PM	TiAlNbSiN-EM
Sa (nm)	34.63 ± 2.17	13.92 ± 2.09
**Coatings**	**AlCrSiN-** **PM**	**AlCrSiN-** **E** **M**
Sa (nm)	72.81 ± 5.5	46.33 ± 5.32

**Table 4:** Hardness and Young’s modulus of TiAlNbSiN and AlCrSiN films.

Coatings	Hardness (GPa)	Young’s Modulus (GPa)	H/E	H^3^/E^2^
TiAlNbSiN				
PM	28.3 ± 1.4	372.4 ± 10.4	0.071	0.144
EM	31.2 ± 1.9	371.0 ± 9.9	0.079	0.194
AlCrSiN				
PM	25.5 ± 0.6	274.2 ± 4.4	0.087	0.195
EM	32.6 ± 0.5	343.6 ± 8.6	0.089	0.257

## Data Availability

The original contributions presented in this study are included in the article. Further inquiries can be directed to the corresponding author.
